# TransMed: Transformers Advance Multi-Modal Medical Image Classification

**DOI:** 10.3390/diagnostics11081384

**Published:** 2021-07-31

**Authors:** Yin Dai, Yifan Gao, Fayu Liu

**Affiliations:** 1 College of Medicine and Biological Information Engineering, Northeastern University, Shenyang 110169, China; daiyin@bmie.neu.edu.cn (Y.D.); yifangao@stumail.neu.edu.cn (Y.G.); 2 Engineering Center on Medical Imaging and Intelligent Analysis, Ministry Education, Northeastern University, Shenyang 110169, China; 3 Department of Oromaxillofacial-Head and Neck Surgery, School of Stomatology, China Medical University, Shenyang 110002, China

**Keywords:** transformer, medical image classification, deep learning, multiparametric MRI, multi-modal

## Abstract

Over the past decade, convolutional neural networks (CNN) have shown very competitive performance in medical image analysis tasks, such as disease classification, tumor segmentation, and lesion detection. CNN has great advantages in extracting local features of images. However, due to the locality of convolution operation, it cannot deal with long-range relationships well. Recently, transformers have been applied to computer vision and achieved remarkable success in large-scale datasets. Compared with natural images, multi-modal medical images have explicit and important long-range dependencies, and effective multi-modal fusion strategies can greatly improve the performance of deep models. This prompts us to study transformer-based structures and apply them to multi-modal medical images. Existing transformer-based network architectures require large-scale datasets to achieve better performance. However, medical imaging datasets are relatively small, which makes it difficult to apply pure transformers to medical image analysis. Therefore, we propose TransMed for multi-modal medical image classification. TransMed combines the advantages of CNN and transformer to efficiently extract low-level features of images and establish long-range dependencies between modalities. We evaluated our model on two datasets, parotid gland tumors classification and knee injury classification. Combining our contributions, we achieve an improvement of 10.1% and 1.9% in average accuracy, respectively, outperforming other state-of-the-art CNN-based models. The results of the proposed method are promising and have tremendous potential to be applied to a large number of medical image analysis tasks. To our best knowledge, this is the first work to apply transformers to multi-modal medical image classification.

## 1. Introduction

Transformers were first applied in the field of natural language processing (NLP) [1]. It is a deep neural network mainly based on the self-attention mechanism to extract intrinsic features of textual data. Because of its powerful representation capabilities, researchers hope to find a way to apply transformers to computer vision tasks. Compared with text, images involve larger size, noise, and redundant modalities, so it is considered more difficult to use transformers on these tasks. Recently, transformers have made a breakthrough in computer vision. A large number of transformer-based methods have been proposed for computer vision tasks, such as DETR [2] for object detection, SETR [3] for semantic segmentation, ViT [4] and DeiT [5] for image classification.

Transformers have achieved success in natural images, but it has received little attention in medical image analysis, especially in multi-modal medical image fusion. Multi-modal images are widely used in medical image analysis to achieve disease classification or lesion segmentation. The existing medical image multi-modal fusion based on deep learning can be divided into three categories: input-level fusion, feature-level fusion, and decision-level fusion [6]. Input-level fusion strategy fuses multi-modal images into the deep network by multi-channel, learns fusion feature representation, and then trains the network. Input-level fusion can retain the original image information to the maximum extent and learn the image features. Feature-level fusion strategy trains a single deep network by taking the image of each modality as a single input. Each representation is fused in the network layer, and the final result is fed to the decision layer to obtain the final result. Feature-level fusion network can effectively capture the information of different modalities of the same patient. Decision-level fusion integrates the output of each network to obtain the final result. Decision-level fusion network aims to learn more abundant information from different modalities independently.

However, they all have shortcomings in varying degrees. The input-level fusion strategy is difficult to establish the internal relationship between different modalities of the same patient, which leads to the degradation of the model performance. Each modality of the feature-level network corresponds to a neural network, which brings huge computational costs, especially in the case of a large number of modalities. The output of each modality of decision-level fusion is independent of each other, so the model cannot establish the internal relationship between different modalities of the same patient. In addition, like decision-level fusion strategy, decision-level fusion strategy is also computationally intensive.

Therefore, there is an urgent need to combine the three fusion strategies efficiently. A good multi-modal fusion strategy should achieve as much interaction between different modalities as possible with low computational complexity.

Compared with CNN, transformers can effectively mine long-range relationships between sequences. The existing computer vision models based on transformer mainly deal with 2D natural images, such as ImageNet [7] and other large-scale datasets. The method of constructing sequences in 2D images is to cut the images into a series of patches. This kind of sequence construction method implicitly shows long-range dependencies, which is not very intuitive, so it may be difficult to bring significant performance improvement.

**Figure 1:**
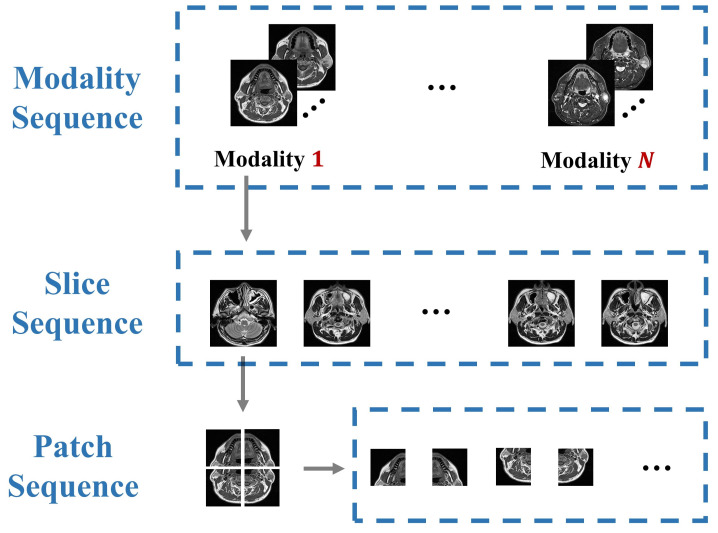
Compared with natural images, multi-modal medical images have more informative sequences.

On the contrary, there are more explicit sequences in medical images, which contain important long-range dependency and semantic information, as shown in Figure 1. Due to the similarity of human organs, most visual representations are orderly in medical images. Destruction of these sequences will significantly reduce the performance of the model. It can be considered that compared with natural images, the sequence relationship of medical images (such as modality, slice, patch) holds more abundant information. In practice, clinicians will synthesize the pathological information of each modality to make the diagnosis. However, most of the existing multi-modal fusion methods do not or rarely consider the correlation of these sequences, and lack of modeling for these long-range dependencies. The transformer structure is an elegant, efficient, and powerful encoder for processing sequence relations, which is the motivation for us to propose the multi-modal medical image classification method based on transformers.

In this work, we present the first study to explore the tremendous potential of transformers in the context of multi-modal medical image classification. The proposed method is inspired by the property that the transformer is effective in extracting the relationship between sequences. However, due to the small scale of medical image datasets and the lack of sufficient information to establish the relationship between low-level semantic features, the performance of pure transformer networks based on ViT and DeiT is not satisfactory in multi-modal medical image classification. Therefore, we propose TransMed, which combines the advantages of CNN and transformer to capture low-level features and cross-modality high-level information. TransMed first processes the multi-modal images as sequences and sends them to CNN, then uses transformers to learn the relationship between the sequences and make predictions. Since the transformer effectively models the global features of multi-modal images, TransMed outperforms the existing multi-modal fusion methods in terms of parameters, operation speed, and accuracy. A large number of experiments have proved the effectiveness of our method.

In summary, we make the following two contributions:

1. We apply transformers to multi-modal medical image classification for the first time and significantly improve the performance of deep models with low computational cost.
2. We propose a novel multi-modal image fusion strategy in this work, which can be leveraged to capture mutual information from images of different modalities in a more efficient way.

The rest of this paper is organized as follows. Section 2 presents some closely related works. The pipeline of our proposed method is in Section 3. Section 4 introduces the experimental results and details. We discuss the results in Section 5. Finally, we summarize our work in Section 6.

## 2. Related Work

### 2.1. Multi-Modal Medical Image Analysis

Multi-modal medical analysis is one of the most fundamental and challenging parts of medical image analysis [8]. It is proved that a reasonable fusion of different modalities has been a potential means to enhance deep networks [6]. Multi-modal fusion can capture more abundant pathological information and improve the quality of diagnosis [9,10].

Some works mainly used the input-level fusion, which is the most common fusion method in multi-modal medical image analysis [11,12,13,14]. Some other papers have shown the potential of feature-level fusion in medical image processing. Hyper DenseNet built dual deep networks for different modalities of Magnetic resonance imaging (MRI) and linked features across these streams [15]. Nie et al. [16] fused final features from modality-specific paths to make final decisions. MMFNet used specific encoders to capture modality-specific features and designs a decoder with a complex structure to fuse these features [17]. Different from the first two techniques, [18,19] applied decision-level fusion technology to improve performance. Tseng et al. [19] designed a novel encoder-decoder structure to capture and fuse low-level and high-level features, then the results of each branch were fused to generate the final result. Shachor et al. [18] set a gate network to dynamically combine each decision and make a prediction.

Besides, some studies have evaluated multiple fusion methods at the same time. Setio et al. [20] used feature-level fusion and decision-level fusion in their work. Guo et al. [21] designed three kinds of fusion networks, and obtains better performance than a single modality. These fusion methods improve the performance of the model to a certain extent, but there are some shortcomings, such as poor scalability, large computational complexity, and difficulty in establishing long-range connections.

### 2.2. Transformers

Transformers were first proposed for machine translation and achieved satisfactory results in a large number of NLP tasks. For a long time, CNN was considered an irreplaceable basic structure in computer vision tasks [22,23,24,25], but currently the breakthrough progress of transformer shows that it is a strong competitor of CNN. Transformers use the self-attention mechanism as the core module to build a convolution-free deep network. Compared with CNN, transformers does not require human-defined inductive bias, and it can handle long-range dependencies well.

Before transformers became popular in the computer vision community, attention and self-attention mechanisms were long used as the auxiliary module of CNN in medical image analysis and greatly improved the performance of deep models. MADGAN [26] integrated the self-attention module into the generative adversarial network for unsupervised medical anomaly detection. Liu et al. [27] developed a CNN with a novel feature pyramid attention mechanism for automatic segmentation of the prostate. Wu et al. [28] proposed a new automated framework that improves the standard U-Net-based architecture through attention modules to accurately delineate epicardial and endocardial contours. Yang et al. [29] presented an advanced segmentation method based on the recursive attention model to segment the left atrium.

The success of attention and self-attention mechanisms motivates researchers to directly apply transformers to computer vision. Some work uses the framework of CNN and transformer [2,30,31], while others directly use pure transformers to replace CNN [2,4,5,32].

Due to its good performance and superiority in capturing long-range dependencies, Transformers have been widely used in medical image segmentation. TransUNet [33] is the first transformer-based medical image segmentation framework, which uses the transformer to encode the global context. CoTr [34] presented a novel framework that efficiently bridges a CNN and a transformer for 3D medical image segmentation. UNETR [35] utilizes a pure transformer as the encoder to effectively capture the multi-scale information.

These methods have shown encouraging results in computer vision tasks and medical image segmentation, but their direct applications in multi-modal medical images are not effective and require a lot of computing resources. As far as we know, TransMed is the first multi-modal medical image classification framework based on transformers, which provides a novel multi-modal image fusion strategy.

## 3. Methods

**Figure 2:**
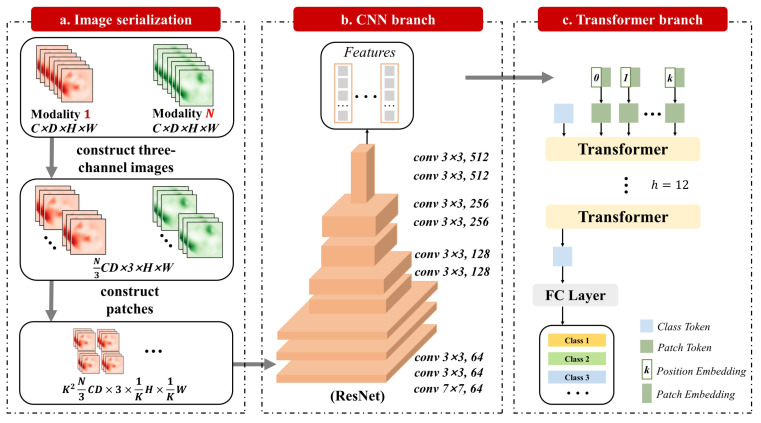
Overview of TransMed, which is composed of CNN branch and transformer branch.

The structure of TransMed is shown in Figure 2. The most common method of multi-modal medical image classification is to train CNN directly (such as Resnet [36]). Firstly, the image is encoded as a high-level feature representation, and then its features or decisions are fused. Different from the existing methods, our method uses transformers to introduce the self-attention mechanism into the multi-modal fusion strategy. We will first introduce how to directly apply transformers to aggregate feature representations from decomposed image patches in Section 3.1. Then, the overall framework of TransMed will be described in detail in Section 3.2.

### 3.1. Transformers Aggregate Multi-Modal Features

In this work, we follow the original DeiT implementation as much as possible. The advantage of this intentionally simple setting is to reduce the impact of other tricks on the performance of the model and intuitively show the benefits of transformers. In addition, we can use the extensible DeiT model and its pre-trained weights almost immediately.

**Figure 3:**
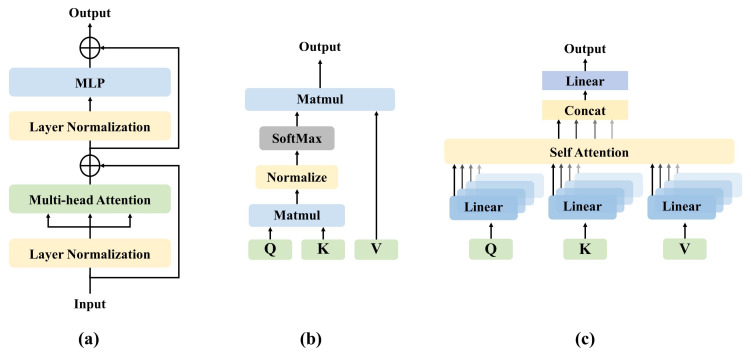
(**a**) Structure of the transformer. (**b**) Overview of self-attention, matmul means matrix product of two arrays. (**c**) An illustration of our multi-head self-attention component, concat means concatenate representations.

The structure of the transformer is shown in Figure 3a. The important components of the transformer including self-attention (SA), multi-head self-attention (MSA), and multi-layer perception (MLP). The input of transformers includes a variety of embeddings and tokens. Slightly different from DeiT, we remove the linear projection layer and distillation token. We will describe each of these components in this section.

#### 3.1.1. Self-Attention

SA is an attention mechanism, which uses other parts of the same sample to predict the rest of the data sample. In computer vision, it is a little similar to non-local networks [37]. SA has many forms, and the common transformer relies on the form of scaled dot-product shown in Figure 3b. In the SA layer, the input vector X is first transformed into three different vectors: query matrix Q, key matrix K, and value matrix V:

(1) $$Q=XW_{q},K=XW_{k},V=XW_{v}$$

where $W_{q}$, $W_{k}$, and $W_{v}$ are trainable matrices. Then, the weight assigned to each value is determined by the dot product of the query and the corresponding key. The attention function between different input vectors is calculated as follows:

(2) $$Attention\left(Q,K,V\right)=Softmax\left(\frac{QK^{T}}{\sqrt{d_{k}}}\right)\cdot V$$

where $d_{k}$ is the dimension of key vector *k*. $\sqrt{d_{k}}$ provides an appropriate normalization to make the gradient more stable.

#### 3.1.2. Multi-Head Self-Attention

MSA is the core component of the transformer. As shown in Figure 3c, the difference from SA is that the multi-head mechanism splits the input into many small parts, then calculates the scaled dot-product of each input in parallel, and splices all the attention outputs to get the final result. The formula of MSA can be written as follows:

(3) $$head_{i}=Attention\left(QW_{i}^{Q},KW_{i}^{K},VW_{i}^{V}\right)$$

(4) $$MSA\left(Q,K,V\right)=Concat\left(head_{1},\ldots ,head_{i}\right)W^{O}$$

where the projections $W_{i}^{Q}$, $W_{i}^{K}$, $W_{i}^{V}$, and $W^{O}$ are trainable parameter matrices; *h* is the number of transformer layers. The advantage of MSA is that it allows the model to learn sequence and location information in different representation subspaces.

#### 3.1.3. Multi-Layer Perceptron

In this paper, an MLP is added on top of the MSA layer. The MLP is composed of linear layers separated by a GeLU [38] activation. Both MSA and MLP have skip-connections like residual networks and with a layer normalization. Therefore, it is assumed that the representation of the t−1 layer is $x_{t-1}$, LN represents the linear normalization, and the output of the *t* layer can be written as follows:

(5) $${\hat{x}}_{t}=MSA\left(LN\left(x_{t-1}\right)\right)+x_{t-1}$$

(6) $$x_{t}=MLP\left(LN\left({\hat{x}}_{t}\right)\right)+{\hat{x}}_{t}$$

#### 3.1.4. Embeddings and Tokens

The input layer contains five embeddings and tokens, which are patch embedding, position embedding, class embedding, patch token, and class token.

Patch embedding is the representation of each patch’s output from CNN, and class embedding is a trainable vector. To encode the spatial information and location information of a patch into patch tokens, we use position embeddings and patch embeddings to preserve the information. Class embedding does not have patch embedding that can be added, so class token and class embedding are equivalent. Suppose the input is *x*, the trainable vector is $W^{c}$, the position embedding is $x_{po}$, patch tokens $x_{pt}$ and class token $x_{ct}$ can be expressed as follows:

(7) $$x_{pt}=Conv\left(x\right)+x_{po}$$

(8) $$x_{ct}=W^{c}$$

The class token is attached to patch tokens before the input layer of transformers, passes through the transformer layer, and then outputs from the fully connected layer to predict the class.

### 3.2. TransMed

Instead of using pure transformers as the encoder, TransMed adopts a hybrid model including CNN and transformer, in which CNN is used as a low-level feature extractor [39] to generate the patch embedding.

Given a multi-modal image $x\in R^{N\times C\times D\times H\times W}$, where spatial resolution is H×W, the depth is *D*, the number of channels is *C*, and the number of modalities is *N*. Before sending it to the CNN encoder, it is necessary to construct the sequence. First, combine the channel dimension, depth dimension, and modality dimension of a multi-modal image to obtain $x^{\prime }\in R^{(N\times C\times D)\times H\times W}$. Then, three adjacent 2D slices of a multi-modal image are superimposed to construct three-channel images $x^{\prime \prime }\in R^{(1/3\times N\times C\times D)\times 3\times H\times W}$. Then, according to [4], each image will be divided into K×K. The larger *K* value means that the size of each patch is smaller. We will evaluate the impact of different *K* values on the performance of the model in Section 4. Finally, the image is encoded into a patch $x_{input}\in R^{\left(1/3\times N\times C\times D\times K^{2}\right)\times 3\times \left(H/K\right)\times \left(W/K\right)}$.

After the image sequence is constructed, it is input into the 2D CNN. The last fully connected layer of 2D CNN is replaced by a linear projection layer to map the features of the vector patch to the potential embedding space. The 2D CNN extracts low-level features from the image sequence and encodes them preliminarily.

## 4. Results

To evaluate the proposed method, we carry out comprehensive experiments on the parotid gland tumor (PGT) dataset and the MRNet dataset. Experimental results demonstrate that TransMed achieves state-of-the-art performance on two datasets. In the following, we first introduce the datasets and preprocessing details. Next, we introduce the experimental settings and evaluation criteria. Then we present a comparison of our model with some state-of-the-arts on the two datasets. Finally, we perform a series of ablation experiments on the PGT dataset.

### 4.1. Dataset

#### 4.1.1. PGT Dataset

**Figure 4:**
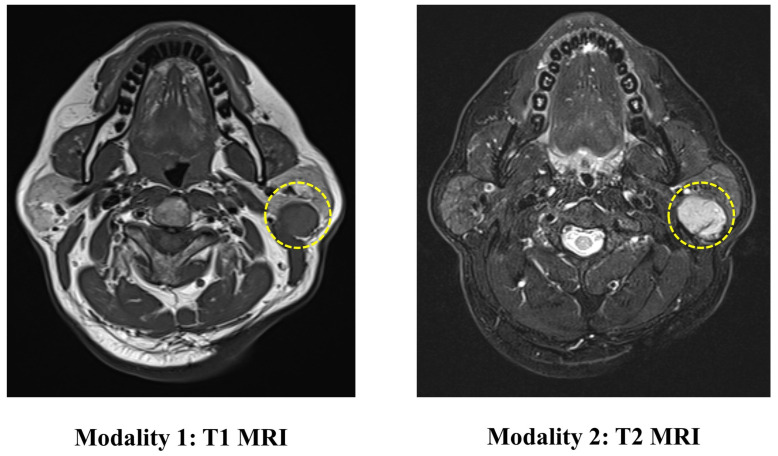
An illustration of the images in the PGT dataset. The yellow circle represents the location of the tumor.

The PGT dataset contains 344 head and neck MRI examinations carried out at the Stomatological Hospital of China Medical University. The ethics board approved the use of the images for this research. This dataset includes two modalities of MRI (T1 and T2), as shown in Figure 4. The ground truth labels are obtained from biopsies.

The incidence of malignant tumors in PGT is about 20% [40]. Correct preoperative diagnosis of these tumors is essential for proper surgical planning. Among them, imaging examination plays an important role in determining the nature of parotid gland masses. MRI is considered to be the preferred imaging method for preoperative diagnosis of PGT [41]. It has been proved in previous studies that in radiation therapy, MRI-based tissue characterization and segmentation are of vital importance for improving the treatment [42,43]. MRI can provide information about the exact location of the lesion, the relationship with the surrounding structure and can assess the spread of nerves and bone invasion. However, it is reported that the PGT shows considerable overlap in imaging features (such as tumor margins, homogeneity, and signal intensity), so it is difficult for doctors to identify the mass.

According to common clinical classifications, we divide PGT into five categories: Pleomorphic Adenoma (PA), Warthin Tumor (WT), Malignant Tumor (MT), Basal Cell Adenoma (BCA), and Other Benign Lesions (OBL) [44]. In the PGT dataset, the patients are randomly divided into the training set (n = 241), validation set (n = 34), and an independent test set (n = 69) according to the ratio of 7:1:2. In this study, we use stratified random sampling to ensure that at least 5 and 10 positive examples of each label are present in the validation and test set, respectively. The training set is used to optimize the model parameters, and the validation set is used to select the best model.

In the data preprocessing stage, we first perform OTSU [45] to extract the foreground area in the original image. Then the images of different modalities of the same patient are registered to improve the consistency of the foreground area. Then resample each image to 18 × 448 × 448. Therefore, each image is a stack of 3D images of MRI T1 and T2, and the size is 36 × 448 × 448. Data augmentation uses random flipping and random noise. Random flipping performs flipping of the image with 50% probability. Random noise adds Gaussian noise with a mean value of 0 and a variance of 0.1 to the image.

#### 4.1.2. MRNet Dataset

The MRNet Dataset contains 1370 knee MRI examinations that were carried out at the Stanford University Medical Center [46]. Each case was labeled according to the anterior cruciate ligament (ACL) tear, meniscus tear, or other signs of abnormalities in the corresponding knee (abnormal). The author randomly split the dataset into 1130 training cases, 120 validation cases, and 120 test cases. The provided dataset includes three modalities of MRI (T1-weighted images, T2-weighted images, and proton density-weighted). Each image is of size 256 × 256 and the number of slices ranges between 17 and 61. The data were preprocessed by applying same procedures used in the MRNet. Data augmentation strategy is consistent with the strategy used in the PGT dataset.

### 4.2. Experimental Settings and Evaluation Criteria

We set SGD as the optimizer with a momentum equal to 0.7. The learning rate is $10^{-3}$, and the maximum training round is 100. The patch size is set to 2. Our experiments were carried out on NVIDIA 3080 GPU. The code is implemented using PyTorch [47] and TorchIO [48]. To eliminate accidental factors, each model is subjected to 10 independent experiments, and other experimental parameters keep consistent during training.

To evaluate the performance of the model, we select the Accuracy (ACC) and Precision (PR) as the evaluation criteria in the PGT dataset. In the MRNet dataset, to produce comparable results with other baseline methods, we use ACC, Area Under Curve (ROC-AUC, or AUC), Sensitivity (SE), and Specificity (SP) as evaluation criteria. TP, TN, FP, and FN are the number of true positive, true negative, false positive, and false negative. ACC is defined as the ratio of the number of correctly classified samples to the total number of samples.

(9) $$ACC=\frac{TP+TF}{TP+TN+FP+FN}$$

SE is defined as calculating the ratio that is correctly classified as positive to those that are true positive samples.

(10) $$SE=\frac{TP}{TP+FN}$$

SP is defined as calculating the ratio that is correctly classified as negative to those that are true negative samples.

(11) $$SP=\frac{TN}{TN+TP}$$

PR is defined as calculating the ratio of samples correctly classified as positive to all samples predicted to be positive.

(12) $$PR=\frac{TP}{TP+FP}$$

AUC is used to evaluate the quality of the binary classification model, which is defined as the area under the ROC curve.

### 4.3. Baseline Methods

#### 4.3.1. PGT Dataset

The input-level fusion strategy and the decision-level fusion strategy can be implemented using mainstream 2D CNN and 3D CNN, so the selected network includes Resnet34, Resnet152, 3D Resnet34, P3D [49], C3D [50], and BoTNet50 [31]. In feature-level fusion experiments, we used two common feature-level fusion methods [15,16]. Since these two papers focus on segmentation tasks, we modify the network structure to adapt to the classification tasks.

#### 4.3.2. MRNet Dataset

In the experiments, we compare our method with three state-of-the-art models: MRNet [46], ELNet [51], and MRPyrNet [52]. MRNet mainly includes three AlexNets [53], which independently make predictions for each modality and use decision-level fusion strategy. ELNet changes the backbone network from AlexNet to Resnet and proposed two technologies: multi-slice normalization and BlurPool layers to improve performance. MRPyrNet uses a Feature Pyramid Network and Pyramidal Detail Pooling to gather and capture small appearing injuries in the knee area. The model was inserted into MRNet and ELNet and achieved significant performance improvement.

### 4.4. Experimental Results

#### 4.4.1. PGT Dataset

Table 1 reports the performance of our proposed models, in which three variants are provided: the tiny version (TransMed-T) use ResNet18 and DeiT-Tiny (DeiT-T) as backbones for CNN branch and transformer branch, respectively; the small version (TransMed-S) use ResNet34 and DeiT-Small (DeiT-S) as backbone; the base version (TransMed-B) uses ResNet50 and DeiT-Base (DeiT-B) as the backbone.

**Figure 5:**
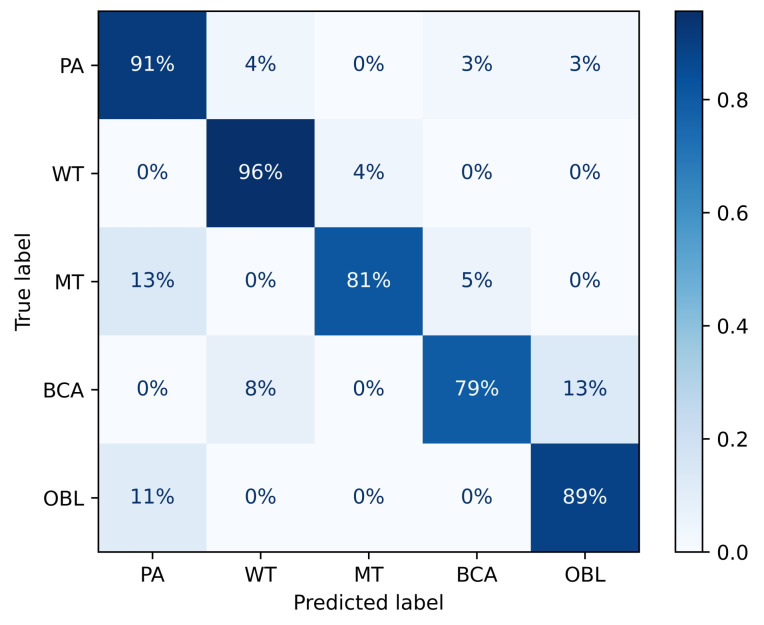
Confusion matrix of TransMed-S on the PGT dataset.

TransMed consistently outperforms previous multi-modal fusion strategies by a large margin. The confusion matrix of TransMed-S is shown in Figure 5, it achieves on average about 10.1% improvement in terms of the average accuracy with respect to the BoTNet, while the larger version TransMed-B slightly suffers from overfitting on the dataset. Table 1 also compares the number of parameters and computational costs between our proposed models and previous methods. TransMed achieves state-of-the-art performance with much fewer parameters and computational costs. TransMed is highly efficient as it models the long-range relationship between modalities very well.

#### 4.4.2. MRNet Dataset

Table 2 reports the performance of MRNet, ELNet, MRPyrNet, and TransMed in the MRNet dataset. Compared with the best performance of the three baseline methods, TransMed achieved 3.5%, 0.5%, and 5.7% improvements in abnormality, ACL tear, and meniscus tear, respectively. Our proposed strategy dramatically improves the abnormality and meniscus tear detection of the baseline method, which shows that the transformer structure can improve the diagnostic ability of the model. More importantly, the proposed method is more robust on the SE, representing a better situation for the potential clinical applications of TransMed. Specifically, compared with the previous techniques, TransMed has achieved a performance improvement of 13.6% (MRPyrNet) to 31.5% (ELNet) in the sensitivity of ACL tear detection.

It is noteworthy that in the MRNet dataset, our method surpasses ELNet [51] and MRPyrNet [52] without inputting any domain knowledge. In ELNet, an experienced radiologist is asked to determine the most informative slice. MRPyrNet strongly assumes that the anomaly is always in the center of the MRI slice.

### 4.5. Ablation Experiments

To demonstrate the effect of transformers in TransMed, we perform ablation experiments on PGT dataset. For TransMed, changing the backbone from TransMed-T to TransMed-S results in 1.9% improvement in average accuracy, at the expense of a much larger computational cost. Therefore, considering the computation cost, all experimental comparisons in this paper are conducted with TransMed-T to demonstrate the effectiveness of TransMed.

In the experiment, TransMed’s CNN and transformers were removed, respectively, and all other conditions remained unchanged. The results are shown in Table 3. The results indicate that the transformer greatly improves the ability of the deep model to explore the relationship between modalities with little increase of parameters and computation. However, the performance of the pure transformer structure is poor due to the small dataset.

We also explored the impact of different patch sizes on performance in image serialization by changing *K* values, respectively, while other conditions remain unchanged. The results are shown in Table 4. The experimental results show that the performance is poor when the *K* value is large. The possible reason is that too small image patches destroy the semantic information of the image.

## 5. Discussion

Mining different sequences of information in multi-modal medical images are essential to improve the performance of deep models. Our work applies transformers to multi-modal medical image classification for the first time because it can effectively explore sequence information that is difficult to capture by CNN. In this work, we first use ResNet to extract the image features and then use Transformer to capture the long-range dependency between the sequences. The experimental results show that our method surpasses the previous state-of-the-art model in the two datasets and has high stability. This study indicates that a hybrid architecture based on CNN and transformers can significantly improve multi-modal medical image classification performance.

To verify the validity of the model, we implement two ablation experiments. The first ablation experiment shows that there is still a big gap between the pure transformer and the typical CNN in small medical image datasets. The reason for the poor performance of the standard vision transformer structure is that the self-attention mechanism does not have an inductive bias similar to the CNN structure. Although when the amount of data is large enough, the transformer structure is proven to surpass the domain knowledge brought by the inductive bias [4]. However, the medical image dataset is small and cannot achieve satisfactory performance.

The second ablation experiment shows that the gain of serializing two-dimensional images is marginal (87.0% vs. 86.8%). Moreover, the model’s performance tends to decline with the increase of *K*. In our work, we follow the existing technology of image serialization in the natural image classification model based on transformers. However, in medical images, such techniques may lead to the degradation of model performance. Because image serialization will separate the tumor, thus the separated tumor area is difficult to recognize as a tumor.

In summary, the preliminary results of our proposed method are encouraging, but there are still many challenges. In order to make the transformer-based structure better used in medical image analysis, it is very important to modify it according to the specific task.

In future work, we will try to overcome the limitations of current research. The first is to apply TransMed to more medical image datasets of different modalities (such as Computed Tomography (CT) and Positron Emission Computed Tomography (PET)) to further investigate the superiority of the proposed model. Moreover, It is necessary to build a robust cross-modality, transformer-based model. Second, we should apply TransMed to other medical image analysis tasks, such as tumor segmentation and lesion detection. Third, we will improve the image serialization technology to further adapt to multi-modal medical images. The fourth is to explore the high-performance structure based on pure transformers. Last but not least, as a fast-developing deep network, the transformer structure requires a reliable and mature visualization technology to improve the interpretability of the model and intuitively illustrate its advantages in capturing long-range dependencies of multi-modal images. Therefore, our future work includes proposing or improving visualization techniques suitable for transformer-based medical image analysis.

## 6. Conclusions

The transformer is a powerful deep neural network structure for processing sequences in NLP, but it has received little attention in medical image analysis. In this paper, we propose TransMed, which is a novel design of multi-modal medical image classification based on transformers. Unlike CNN-based methods, TransMed uses a hybrid model including CNN and transformer. Among them, CNN is used as a low-level feature extractor to generate local feature sequences of multi-modal images; while transformers effectively extract long-range dependencies between sequences from low-level feature sequences to achieve good performance. In the two multi-modal medical image classification datasets, our method achieved an average accuracy improvement of 10.1% and 1.9%, respectively, compared with the previous state-of-the-art models. Our experiments provided insights on the inclusion of transformers in deep networks for medical image analysis, particularly in multi-modal scenarios. Combining these promising results, we believe that the transformer structure has tremendous potential in a large number of medical image analysis tasks.

## Figures and Tables

**Table 1:** Comparison on the PGT dataset (average ACC % and PR % for each disease. IF, FF, and DF represent input-level fusion, feature-level fusion and decision-level fusion, respectively). All the results are presented as mean ± standard deviation. The throughput is calculated as the time consumption (second) of predicting 100 images, which is measured using 2080Ti GPU.

Method	Dim	Fusion	Params	Throughput	ACC	PA	WT	MT	BCA	OBL
P3D	3D	IF	67 M	15.8	76.1 ± 5.5	59.9 ± 23.1	84.3 ± 5.3	69.7 ± 19.0	71.4 ± 7.3	78.0 ± 14.0
C3D	3D	IF	28 M	32.3	71.0 ± 4.1	68.3 ± 38.9	81.3 ± 15.4	67.8 ± 10.0	71.4 ± 7.5	84.5 ± 12.4
Resnet34	2D	IF	22 M	4.0	69.9 ± 4.0	81.0 ± 11.0	77.6 ± 7.4	61.4 ± 10.8	53.8 ± 15.5	68.1 ± 7.9
Resnet152	2D	IF	58 M	6.3	69.0 ± 3.5	50.5 ± 18.0	74.1 ± 10.1	64.3 ± 20.0	62.9 ± 8.9	75.2 ± 11.4
3D Resnet34	3D	IF	64 M	23.8	73.3 ± 5.1	69.2 ± 16.2	86.8 ± 5.2	75.3 ± 18.1	68.7 ± 13.8	65.7 ± 6.4
BoTNet50	3D	IF	21 M	4.2	77.4 ± 2.0	82.6 ± 14.4	84.0 ± 4.1	76.1 ± 6.9	76.0 ± 12.4	70.8 ± 8.4
[15]	3D	FF	45 M	27.9	74.2 ± 2.9	76.0 ± 24.8	86.2 ± 10.0	72.9 ± 16.3	75.2 ± 24.6	80.0 ± 15.2
[16]	3D	FF	130 M	30.8	73.3 ± 2.4	46.2 ± 13.2	78.4 ± 7.9	70.2 ± 15.5	69.8 ± 15.8	79.0 ± 10.7
P3D	3D	DF	136 M	22.1	74.8 ± 4.6	50.5 ± 20.0	85.1 ± 4.4	70.5 ± 20.2	69.5 ± 8.7	73.4 ± 14.3
C3D	3D	DF	57 M	41.3	71.0 ± 3.3	58.3 ± 33.3	70.7 ± 9.3	74.0 ± 8.5	78.9 ± 20.0	73.2 ± 6.7
Resnet34	2D	DF	45 M	5.6	71.3 ± 4.5	72.7 ± 21.7	75.3 ± 8.1	72.5 ± 10.3	60.9 ± 16.8	70.3 ± 9.7
Resnet152	2D	DF	116 M	9.5	72.2 ± 5.5	63.5 ± 18.3	75.6 ± 10.4	73.4 ± 18.7	83.2 ± 16.3	69.6 ± 11.5
3D Resnet34	3D	DF	128 M	34.6	72.1 ± 3.5	64.7 ± 14.4	81.5 ± 9.3	66.8 ± 8.4	69.6 ± 8.9	72.1 ± 14.9
BoTNet50	3D	DF	44 M	6.6	78.8 ± 3.4	72.6 ± 6.2	82.9 ± 4.3	73.2 ± 8.2	76.9 ± 16.2	87.9 ± 8.2
TransMed-T	2D	——	17 M	4.2	87.0 ± 2.6	80.1 ± 13.8	87.3 ± 3.0	90.7 ± 5.1	82.5 ± 15.3	**93.6 ± 3.3**
**TransMed-S**	2D	——	43 M	4.5	**88.9 ± 3.0**	**90.1 ± 12.2**	**89.2 ± 6.8**	**92.0 ± 4.4**	82.9 ± 9.3	88.3 ± 6.1
TransMed-B	2D	——	110 M	6.3	87.4 ± 2.1	86.2 ± 15.2	88.4 ± 3.8	88.2 ± 7.0	**84.8 ± 13.8**	92.2 ± 8.0

**Table 2:** Comparison on the MRNet dataset.

Pathology	Method	ROC-AUC	ACC	SE	SP
Abnormality	MRNet	0.936	0.883	0.947	0.64
	ELNet	0.941	0.917	**0.968**	0.72
	MRPyrNet (with MRNet)	–––	–––	–––	–––
	MRPyrNet (with ELNet)	–––	–––	–––	–––
	TransMed-T (Ours)	0.974 ± 0.007	0.907 ± 0.010	0.955 ± 0.002	**0.728 ± 0.016**
	TransMed-S (Ours)	**0.976 ± 0.004**	**0.918 ± 0.006**	0.958 ± 0.011	0.720 ± 0.000
	TransMed-B (Ours)	0.958 ± 0.018	0.898 ± 0.012	0.951 ± 0.016	0.696 ± 0.020
ACL Tear	MRNet	0.955 ± 0.005	0.847 ± 0.005	0.722 ± 0.000	**0.950 ± 0.009**
	ELNet	0.940 ± 0.001	0.808 ± 0.000	0.648 ± 0.019	0.939 ± 0.015
	MRPyrNet (with MRNet)	0.976 ± 0.003	0.886 ± 0.010	0.815 ± 0.019	0.944 ± 0.009
	MRPyrNet (with ELNet)	0.960 ± 0.015	0.881 ± 0.034	0.827 ± 0.039	0.924 ± 0.030
	TransMed-T (Ours)	0.969 ± 0.009	0.938 ± 0.009	0.935 ± 0.021	0.939 ± 0.008
	TransMed-S (Ours)	**0.981 ± 0.011**	**0.949 ± 0.003**	**0.963 ± 0.007**	0.938 ± 0.005
	TransMed-B (Ours)	0.949 ± 0.013	0.931 ± 0.012	0.924 ± 0.027	0.936 ± 0.006
Meniscus Tear	MRNet	0.843 ± 0.016	0.778 ± 0.027	0.750 ± 0.067	0.799 ± 0.009
	ELNet	0.869 ± 0.031	0.775 ± 0.044	0.814 ± 0.109	0.745 ± 0.075
	MRPyrNet (with MRNet)	0.889 ± 0.006	0.808 ± 0.008	0.853 ± 0.048	0.775 ± 0.052
	MRPyrNet (with ELNet)	0.895 ± 0.008	0.761 ± 0.042	0.872 ± 0.106	0.676 ± 0.149
	TransMed-T (Ours)	0.939 ± 0.015	0.830 ± 0.024	0.869 ± 0.018	0.800 ± 0.032
	TransMed-S (Ours)	0.945 ± 0.011	0.848 ± 0.016	**0.881 ± 0.037**	0.824 ± 0.026
	TransMed-B (Ours)	**0.952 ± 0.012**	**0.853 ± 0.018**	0.879 ± 0.039	**0.834 ± 0.007**

**Table 3:** Ablation study on the effectiveness of CNN branch and transformer branch.

Model	Params	TFlops	Acc	PA	WT	MT	BCA	OBL
TransMed-T	17 M	0.09	87.0 ± 2.6	80.1 ± 13.8	87.3 ± 3.0	90.7 ± 5.1	82.5 ± 15.3	93.6 ± 3.3
w/o transformer	12 M	0.01	71.3 ± 2.5	71.6 ± 17.8	78.9 ± 3.9	73.9 ± 12.5	66.9 ± 13.1	69.9 ± 13.3
w/o CNN	5 M	0.07	51.3 ± 5.9	20.0 ± 18.7	61.5 ± 13.5	37.0 ± 5.7	41.7 ± 40.1	51.0 ± 16.4

**Table 4:** Ablation study on different patch sizes.

Model	K	ACC	PA	WT	MT	BCA	OBL
TransMed-T	1	86.8 ± 2.3	83.1 ± 12.4	90.1 ± 3.9	88.7 ± 10.1	78.3 ± 13.7	95.3 ± 5.9
TransMed-T	2	87.0 ± 2.6	80.1 ± 13.8	87.3 ± 3.0	90.7 ± 5.1	82.5 ± 15.3	93.6 ± 3.3
TransMed-T	4	86.4 ± 3.3	86.3 ± 17.2	87.0 ± 6.5	89.5 ± 3.6	75.5 ± 8.3	92.1 ± 7.2
TransMed-T	8	80.0 ± 5.8	81.2 ± 17.4	81.1 ± 5.3	88.5 ± 4.2	63.9 ± 12.1	88.2 ± 13.6
TransMed-T	16	65.2 ± 5.8	49.0 ± 32.2	72.1 ± 7.7	70.4 ± 7.9	62.3 ± 23.0	64.1 ± 14.5

## Data Availability

The data presented in this study are available on request from the corresponding author.
